# Ultrasonographic classification of 26 cases of fetal umbilical-portal-systemic venous shunts and the correlations with fetal chromosomal abnormalities

**DOI:** 10.1186/s12884-023-05525-5

**Published:** 2023-04-10

**Authors:** Lihua Lu, Limin Yao, Hui Wei, Jilin Hu, Dongmei Li, Yifei Yin, Jie Su, Qian Li, Shu Zhu, Xinhua Tang, Wenming Huang, Baosheng Zhu, Jinman Zhang

**Affiliations:** 1 Faculty of Environmental Science and Engineering, Kunming University of Science and Technology, Kunming, Yunnan 650500 P.R. China; 2 National Health Commission Key Laboratory of Preconception Health Birth in Western China, Yunnan Provincial Clinical Research Center for Birth Defects and Rare Diseases, Department of Obstetrics and Gynecology, Affiliated Hospital of Kunming University of Science and Technology, The First People’s Hospital of Yunnan Province, Kunming, Yunnan 650032 P.R. China; 3 Department of Medical Ultrasonics, People’s Hospital of Guangxi Zhuang Autonomous Region, Guangxi Academy of Medical Sciences, Nanning, Guangxi 530021 P.R. China; 4 Department of Medical Ultrasonics, Prenatal Diagnosis Center of Guangxi Zhuang Autonomous Region, Maternity and Child Health-care Hospital of Guangxi Zhuang Autonomous Region, Nanning, Guangxi 530028 P.R. China

**Keywords:** Umbilical-portal-systemic venous shunt, Prenatal ultrasonography, Structural malformations, Chromosomal abnormalities

## Abstract

**Objective:**

To investigate the ultrasonographic classification of fetal umbilical-portal-systemic venous shunts (UPSVS) and the correlations with fetal chromosomal abnormalities.

**Methods:**

We retrospectively analyzed the ultrasound characteristics and the corresponding chromosomal abnormalities of 26 cases of fetal UPSVS prenatally diagnosed.

**Results:**

A total of 26 fetuses diagnosed as UPSVS were included, including four cases of type I UPSVS, ten of type II, three of type IIIA, and nine of type IIIB. Four cases of type I were all complicated by fetal heart enlargement and heart insufficiency, of which one case had multiple malformations, and all four cases terminated pregnancies. Six of ten cases of type II terminated pregnancies, including four of Down’s syndrome, one of twin reversed arterial perfusion sequence, one of fetal edema but with normal copy number variation (CNV) by chorionic villus sampling. The other four of ten cases were isolated type II with normal chromosomes, which were delivered at full term and were normal in growth and development when followed up 34 months after birth. Three cases of type IIIA all terminated pregnancies, of which one had multiple malformations, one had right multicystic dysplastic kidney, and one had fetal heart enlargement and heart failure. Among nine of type IIIB, seven with chromosomal abnormalities and/ or complicated malformations terminated pregnancies, and two with isolated type IIIB and normal chromosomes were delivered at full term, and were normal in growth and development (one was followed up to 33 months after birth and the other 20 months after birth).

**Conclusion:**

Fetal UPSVS can be clearly diagnosed and typed by prenatal ultrasonography. Fetal prognosis is determined by the types of UPSVS and complicated malformations and/ or chromosomal abnormalities. The probability of fetal chromosomal abnormalities in UPSVS fetuses is related to the ultrasonographic classification.

## Introduction

Fetal umbilical-portal-systemic venous shunts (UPSVS) refers to abnormal venous communication between portal vein and systemic vein, which is a low-incidence congenital vascular malformation [1–4]. UPSVS is classified into four types: type I: umbilical-systemic shunts (USS), type II: ductus venosus-systemic shunts (DVSS), type IIIA: intrahepatic portal-systemic shunts (IHPSS), and type IIIB: extrahepatic portal-systemic shunts (EHPSS) [1]. The direct entry of venous blood from umbilical and portal veins into systemic venous system leads to inadequate intrahepatic portal venous blood perfusion and portal-systemic shunt, and consequently, fetal heart failure, fetal hydrops and fetal growth restriction (FGR) in the fetal period [1, 4–6]. Postnatal clinical manifestations may include different degrees of liver impairment, metabolic abnormalities, and even hepatic encephalopathy, pulmonary arterial hypertension and hepatopulmonary syndrome [6–10]. Clinical manifestations of UPSVS are diverse. Fetal prognosis may be determined by the types of UPSVS and the complicated structural malformations and/ or chromosomal abnormalities [1, 3, 6, 11]. The incidence rate of UPSVS in neonatal period was reported to be 1/30 000–1/25 000, and that in fetal period has not been reported yet [1, 5]. UPSVS was associated with fetal chromosomal abnormalities, whereas gene syndromes have not been reported [1–3].

In this study, we analyzed the ultrasound characteristics, genetic test results, pregnancy outcomes and neonatal prognosis of 26 cases of UPSVS, in order to explore the ultrasonographic classification of fetal UPSVS and its correlations with fetal chromosomal abnormalities. The deeper understanding of fetal UPSVS may provide more comprehensive basis for prenatal consultation and postnatal management.

## Materials and methods

### Study patients

A total of 26 cases diagnosed as fetal UPSVS by ultrasonography in the Department of Medical Genetics, the First People’s Hospital of Yunnan Province, China, from August 2019 to August 2021, were collected as study patients, of which twenty-one cases were naturally conceived (twenty singleton pregnancies, one twin pregnancy with one UPSVS fetus and the other acarida), five cases were conceived with assisted reproductive technology (two singleton pregnancies, three twin pregnancies all with one UPSVS fetus). The range of maternal age was 21–42 years (average 31.5 ± 6.6 years). The gestational weeks at the diagnosis of UPSVS were 12–28 weeks (average 20.6 ± 4.7 weeks).

### Methods

#### Ultrasonic instrument

Color Doppler ultrasonic instrument (Voluson E10, GE Company, USA), abdominal convex array probe with frequency range 2.5–5.0 MHz.

#### Examination method

Fetal systematic ultrasound examinations were performed following the Guidelines for Prenatal Ultrasound Examination by the Chinese Doctors Association Sonographer Branch (2012) [12]. The tri-plane scanning method proposed by Yagel, et al. was applied to observe fetal umbilical-portal-systemic veins (UPSV) [13]. UPSV includes umbilical vein (UV), portal vein (PV), and ductus venous (DV) [1]. UPSVS was suspected when abnormal course of UV, DV, or intrahepatic portal vein (HPV) and its branches were observed. Subsequently, detailed ultrasound scan was performed to observe the courses and blood flow of fetal UV, HPV and hepatic vein (HV), to assess the development of HPV and its branches, and to observe the lumen, blood filling and flow of main portal vein (MPV) and hepatic artery (HA) at the first porta hepatica. The course of splenic vein (SV) and its confluence were observed from splenic hilum. The course of superior mesenteric vein (SMV) and its confluence were observed along the superior mesenteric artery (SMA). If abnormal communicating branches or abnormal drainage veins was observed, the starting and ending parts of the vessels and their courses were further observed. UPSVS was classified according to the method by Achiron et al. [1].

#### Ultrasound reexaminations at follow-up visits

At the first time when UPSVS was found, an echocardiography was performed for each fetus. For women who continued pregnancies, we tried our best to conduct fetal ultrasonography in follow-up visits according to the cardiovascular profile score (CVPS) system[14] which was used to evaluate fetal cardiovascular function and determine the interval for next reexamination. CVPS = 10 points, reexamined after 4 weeks; 10 > CVPS ≥ 7 points, reexamined after 2 weeks; 7 > CVPS ≥ 5 points, reexamined after 1 week; CVPS < 5 indicated poor fetal outcome. However, if the conditions did not permit, it was usually recommended to have follow-up visit every 4 weeks. At each follow-up visit, fetal systematic ultrasonography was performed, in which the fetal heart was carefully observed, and an additional echocardiography was performed if necessary. However, with the increase of gestational age, obstruction of fetal ribs to ultrasound in late pregnancy often made the performance of echocardiography difficult, so that we sometimes could not finish a complete echocardiography.

#### Fetal karyotype analysis and copy number variation sequencing (CNV-Seq)

Invasive prenatal diagnosis was performed after informed consent. Villus/ amniotic fluid samples were collected for cell culture-karyotype analysis and CNV-Seq for genetic tests.

#### Follow-up of pregnancy outcomes

Based on ultrasonographic types of UPSVS, results of karyotype analysis and CNV-Seq, genetic counseling or multidisciplinary consultation was conducted. Subsequently, the couples made informed choice whether to continue pregnancies. For the continued pregnancies, pregnancy outcomes were followed up by telephone. Until December 2022, the maximum age of live birth baby was 33 months.

### Statistics

Data were statistically analyzed using SPSS software (version 20.0, IBM Corp., Armonk, New York, USA). Continuous variables with normal or non-normal distribution were recorded as “mean ± standard deviation” (mean ± SD) or “median (interquartile range)”. Categorical variables were recorded as frequencies and percentages. For continuous variables, normal distribution data were compared by analysis of variance (ANOVA) or Welch’s ANOVA (heterogeneity of variance), and Bonferroni post-hoc test was used for the comparison between two groups. Non-normal distribution data were analyzed using Kruskal-Wallis test and Dunn’s multiple comparisons. Tests for categorical variables were applied based on expected frequencies. P < 0.05 was considered statistically significant.

## Result

For the 26 cases of UPSVS in this study, the earliest gestational weeks at diagnosis was 12 weeks, and the latest was 28 weeks, with an average of 21 weeks. Among them, ten cases (38%) had advanced maternal age (≥ 35 years), and fourteen cases (54%) had advanced paternal age (≥ 35 years).

Clinical data of the 26 cases of UPSVS were shown in Table 1. Among them, twenty cases terminated pregnancies, and six continued pregnancies to term deliveries; twenty received invasive prenatal diagnosis, and nine had fetal chromosomal abnormalities. According to the classification method of UPSVS put forward by Achiron, et al., four cases (15%) were diagnosed as type I, ten (38%) were type II, three (12%) were type IIIA, and nine (35%) were type IIIB [5]. All four cases of type I manifested as fetal heart enlargement and heart failure. Four of ten cases of type II had chromosomal abnormalities (all four were trisomy 21). Among the ten cases of type III who received invasive prenatal diagnosis, five cases of type IIIB had fetal chromosomal abnormalities (two of trisomy 18, one of trisomy 3 mosaicism, one of chromosome 10 micro-deletion, one of chromosome 8 micro-duplication). In UPSVS fetuses, type II and type IIIB were mostly complicated by chromosomal abnormalities, which were 44% (4/9) and 50% (5/10), respectively.

**Table 1:** Ultrasonographic classification, invasive prenatal diagnosis and fetal outcomes of 26 cases of UPSVS

Ultrasonographic classification	Cases of each type (n)	Cases with complicated malformations (n)	Cases received invasive prenatal diagnosis (n)	Results of prenatal diagnosis		Cases terminated pregnancies (n)	Cases of live births (n)
				Aneuploidy and the mosaic (n)	Copy number variations (n)		
I	4	1	1	0	0	4	0
II	10	0	9	4	0	6	4
IIIA	3	2	2	0	0	3	0
IIIB	9	2	8	3	2	7	2
Total	26	5	20	7	2	20	6

**Fig. 1:**
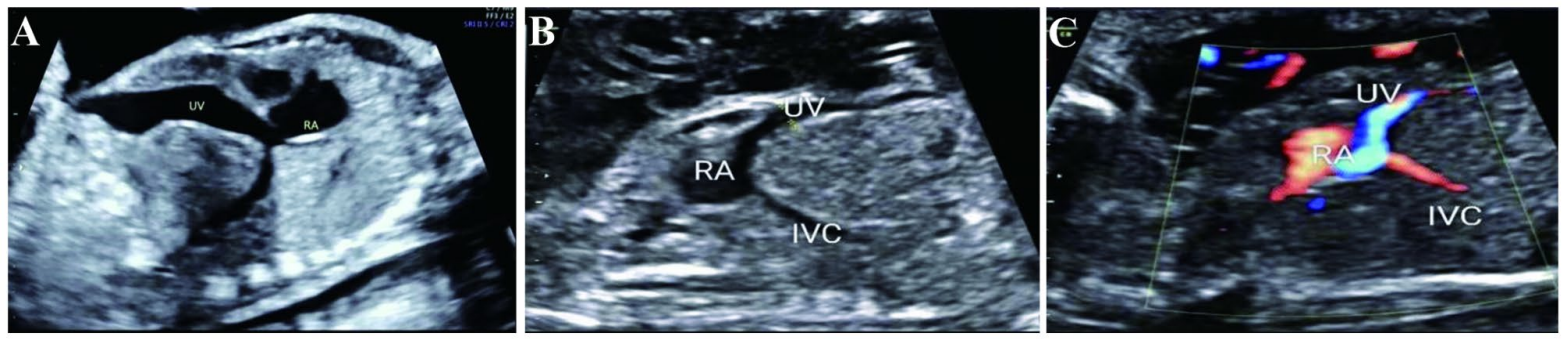
Ultrasonic images of type I UPSVS (umbilical vein-systemic shunt, USS) (**A**-**C**) The umbilical vein (UV) directly connected with the right atrium (RV)

In order to show the frequency of UPSVS in our center, we made statistics on frequency of other structural anomalies in our center in the corresponding period, as shown in Table 2. In this study, the anatomic abnormality that umbilical vein runs on the liver surface and directly connects with right atrium was observed in all four cases of type I (umbilical vein-systemic shunt, USS), as shown in Fig. 1. Meanwhile, the UV-LPV-DV complex was not detected in fetal liver, and intrahepatic portal vein was unclear on ultrasonography. Due to anatomic abnormality, the umbilical vein blood flowed directly into the right atrium without entering the liver, which led to significantly increased blood volume load of the right atrium. This could be the reason why all four cases of type I manifested as heart enlargement and heart failure, and all terminated pregnancies due to the subsequent poor fetal prognosis.

**Table 2:** Frequency of structural anomalies in the corresponding period

Structural anomaly	cases(n)	frequency (%)
UPSVS	26	2.84
Cardiac malformations	246	26.91
Multiple malformations	195	21.33
Urinary system	92	10.07
Hydramnios/oligoamnios	79	8.64
Skeletal system	49	5.36
Strephenopodia	37	4.05
Respiratory system	32	3.50
Cleft lip and/or palate	30	3.28
Nervous system	28	3.06
Fetal edema	28	3.06
Digestive system	27	2.95
Auricle deformity	13	1.42
Limbs	10	1.09
Acromphalus	9	0.98
Situs inversus	4	0.44
Genital system	4	0.44
Diaphragmatic hernia	3	0.33
Open neural tube defects	2	0.22
Total	914	100

**Fig. 2:**
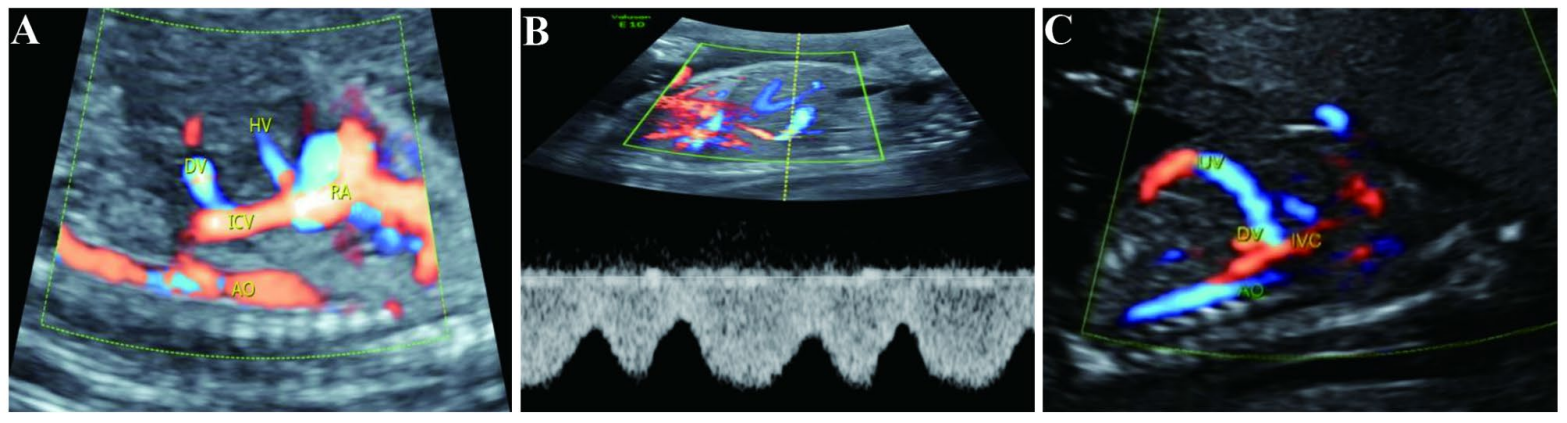
Ultrasonic images of type II UPSVS (ductus venous-systemic shunt, DVSS) (**A**-**C**) The UV-LPV-DV complexes shifted down, and the ductus venosus (DV) did not connect to subdiaphragmatic vestibule, but ectopically connected to the liver segment of inferior vena cava (IVC)

The ten cases of type II (ductus venous-systemic shunt, DVSS) all had intact UV-LPV-DV complexes. However, the ductus venosus did not connect to subdiaphragmatic vestibule, but ectopically connected to the liver segment of inferior vena cava, as shown in Fig. 2. Among cases of type II, six of ten cases terminated pregnancies. Four cases were invasively diagnosed as trisomy 21, with complicated structural malformations as aberrant right subclavian artery, ventricular septal defect, nuchal fold thickening, absence of ossified nasal bone, echogenic bowel, and FGR. One case was monochorionic diamniotic twins, in which fetus A was type II UPSVS and fetus B was acardia malformation, but the couple declined prenatal diagnosis. One case had normal karyotyping and CNV-seq results, but complicated by fetal edema, nuchal translucency thickening, and cervical cystic hygroma. Four of ten cases had isolated type II UPSVS and normal karyotyping and CNV-seq results, and continued pregnancies to term deliveries. The four babies were normal in growth and development in the follow-ups at 33 months after birth.

**Fig. 3:**
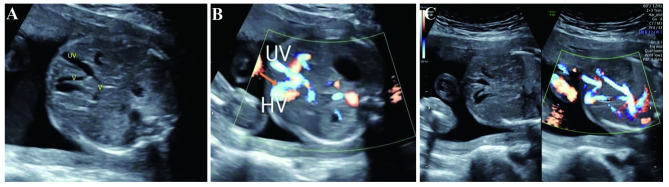
Ultrasonic images of type IIIA UPSVS (intrahepatic portal-systemic shunt, IHPSS) (**A**-**C**) A communicating branch connected the left portal vein with the middle hepatic vein. The arrow showed the communicating branch. *UV*: umbilical vein, *HV*: hepatic vein

All the three cases of type IIIA (intrahepatic portosystemic shunt, IHPSS) had complicated structural malformations, and all terminated pregnancies. One case had a communicating branch connecting left portal vein (LPV) and middle hepatic vein, absence of UV-LPV-DV complex, complete transposition of the great arteries, right aortic arch, absence of right lung, right ureter stenosis near the bladder, severe right hydronephrosis, and single umbilical artery, as shown in Fig. 3. One case had a communicating branch connecting right branch of portal vein and right hepatic vein, and right multicystic dysplastic kidney. One case had a communicating branch connecting left lateral lobe portal vein and left hepatic vein, fetal heart enlargement (heart-chest area ratio = 0.44), and mitral and tricuspid regurgitation. Two of them received prenatal diagnosis, and had normal karyotyping and CNV-seq results.

**Fig. 4:**
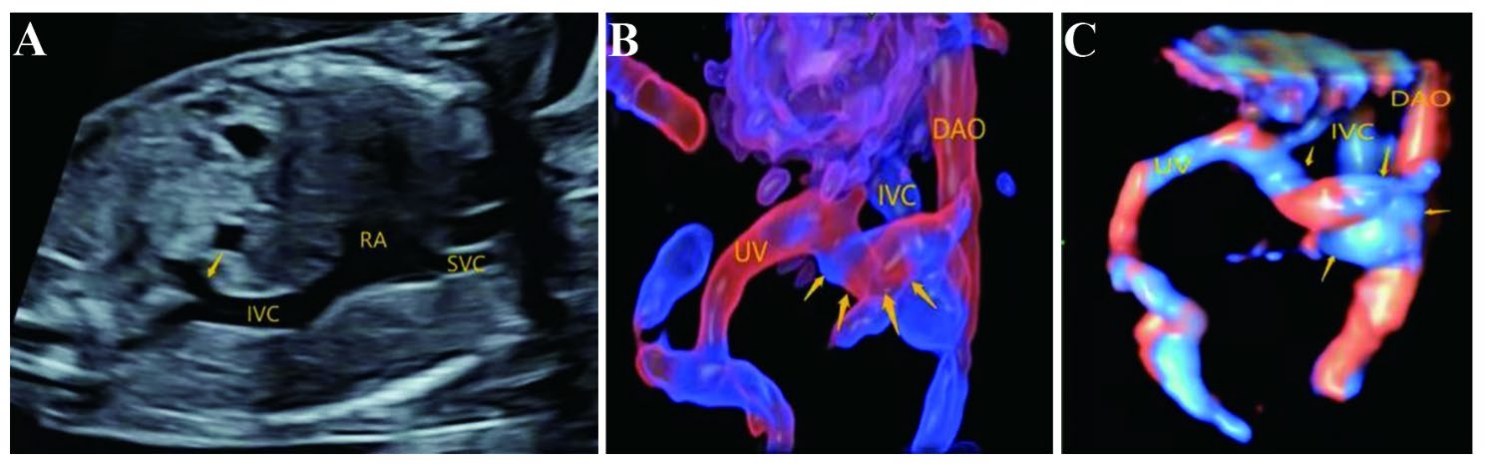
Ultrasonic images of one case of type IIIB UPSVS (extrahepatic portal-systemic shunts, EHPSS) (**A**-**C**) An abnormal blood vessel originated from the portal sinus, tortuously coursed to the right, posterior and inferior, and merged into the hepatic segment of inferior vena cava (IVC). The arrow showed the abnormal blood vessel. (**B**) was obtained by 3D HD-live flow silhouette, and (**C**) by 3D HD-live flow

**Fig. 5:**
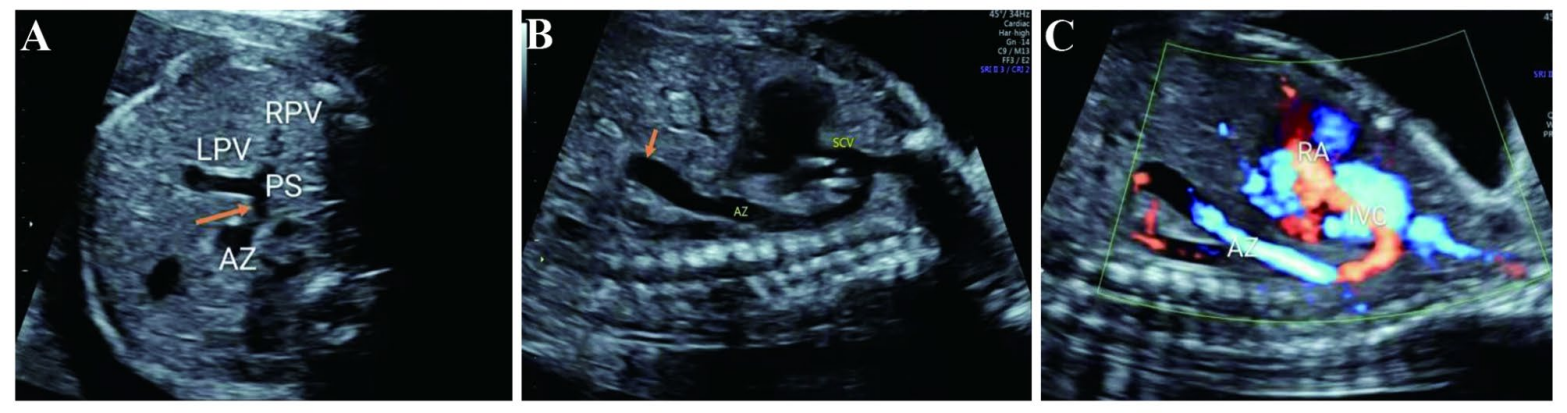
Ultrasonic images of another case of type IIIB UPSVS (extrahepatic portal-systemic shunts, EHPSS) (**A**-**C**) An abnormal blood vessel originated from the portal sinus, tortuously coursed to the left, posterior and upper, and merged into the azygos vein

The nine cases of type IIIB (extrahepatic portal-systemic shunts, EHPSS) had common ultrasound characteristics that main portal vein blood flow into the liver was not detected at the first porta hepatis, and the hepatic artery was widened compensatorily. In seven of the nine cases, UV-LPV-DV complex was detected. one case had an abnormal blood vessel originating from portal sinus, tortuously coursing to the right, posterior and inferior, and merging into hepatic segment of inferior vena cava, as shown in Fig. 4. Fetal complicating structural abnormalities included FGR, ventricular septal defect, coarctation of aortic arch, bulging of right diaphragm, and bilateral choroid plexus cysts. The karyotyping result was trisomy 18. One case had abnormal left-hand posture, bilateral choroid plexus cysts, and abnormal karyotype of trisomy 18. One case had absence of ossified nasal bone and abnormal karyotype of 47,XN,+3(8) / 46,XN(44) mosaic. One case had fetal edema, thickened nuchal fold of 7.7 mm, but the couple declined invasive prenatal diagnosis. The other three cases had normal inner diameter of venous duct, well-filled intrahepatic portal vein, and none of other structural abnormalities. Among them, one case had an abnormal blood vessel originating from portal sinus, tortuously coursing to the left, posterior and inferior, and merging into left internal iliac vein; one case had an abnormal blood vessel originating from portal sinus, tortuously coursing to the left, posterior and upper, and merging into the azygos vein, as shown in Fig. 5. These two cases declined invasive prenatal diagnosis, and continued pregnancies to term deliveries. Follow-ups until December 2022, 20 and 33 months after birth respectively, showed that one baby was normal in growth and development, and the other had elevated total bile acid of 31 µmol/L (normal reference range 0–15 µmol/L), while other indicators of liver function test were normal. She has received ursodeoxycholic acid treatment. At present, she had normal intelligence development and a normal body weight of 13 kg and height of 84 cm. However, these two cases of type IIIB UPSVS did not have angiography to confirm the congenital vascular malformations. These two cases were also the only two live births among nine cases of type IIIB. The other case had an abnormal blood vessel originating from portal sinus, tortuously coursing to the right, posterior and inferior, merging into liver segment of inferior vena cava. CNV-Seq result showed an micro-duplication of 46,XY, dup(8)(p23.1), 3.8 Mb, and the pregnancy was terminated by induced labor. Two of nine cases of type IIIB had no UV-LPV-DV complex, complicated with absence of intrahepatic intrinsic portal vein, absence of main portal vein and its blood flow at the first porta hepatis, enlarged hepatic artery, and abnormal intrahepatic portal vein and its branches. One of the cases had normal karyotyping and CNV-Seq results, but the couple chose to terminate pregnancy. The other case was dichorionic diamniotic twins conceived by IVF-ET technology. Fetus A was type IIIB UPSVS, and resulted in stillbirth at 26 gestational weeks. Fetus B was normal on ultrasonography, delivered at full term, and was normal in growth and development in the follow-up at 4 months after birth. Both twin fetuses had normal karyotyping and CNV-Seq results.

The ultrasonographic characteristics, including the complicated cardiac and extracardiac anomalies, CVPS scoring, karyotype and CNV-Seq results, and pregnancy outcomes of the 26 UPSVS fetuses were summarized in Table 3, and the frequency of UPSVS coexistent defects/ anomalies in Table 4.

**Table 3:** Ultrasonographic characteristics and pregnancy outcome of 26 UPSVS fetuses

No.	Type of UPSVS	Complicated cardiac anomalies	CVPS scoring	Complicated extracardiac anomalies	Karyotype and CNV-Seq	Fetal outcomes
1	I	-	5	SUA	-	Terminated
2	I	-	7	-	N/A	Terminated
3	I	-	6	-	N/A	Terminated
4	I	VSD	5	CDH	N/A	Terminated
5	II	ARSA	9	-	47,XN,+21	Terminated
6	II	-	10	-	47,XY,+21	Terminated
7	II	VSD	9	FGR, ANB	47,XN,+21	Terminated
8	II	-	10	-	-	Term birth
9	II	-	10	-	-	Term birth
10	II	-	10	-	-	Term birth
11	II	-	5	-	47,XN,+21	Stillbirth
12	II	-	10	TRAPs	-	Terminated
13	II	-	6	AHP	N/A	Terminated
14	II	-	10	-	-	Term birth
15	IIIA	TGA, RAOA	10	RPD, HN, SUA	N/A	Terminated
16	IIIA	-	10	R-MCDK	-	Terminated
17	IIIA	-	6	-	-	Terminated
18	IIIB	-	4	FGR, ANB	47,XN,+3(8)/46,XN(44)	Terminated
19	IIIB	-	10	-	N/A	Term birth
20	IIIB	-	10	SUA	46,XX,del(10)(q26.37)	Terminated
21	IIIB	-	10	-	-	Stillbirth
22	IIIB	-	10	-	46,XY,dup(8)(p23.1)	Terminated
23	IIIB	-	5	FE	N/A	Terminated
24	IIIB	-	8	AHP, CPC	47,XY,+18	Terminated
25	IIIB	VSD	9	CPC	47,XY,+18	Terminated
26	IIIB	-	10	-	N/A	Term birth

“-”: normal test/ ultrasonographic result

N/A: Karyotype and CNV-Seq were not performed

*VSD*: ventricular septal defect; *CDH*: congenital diaphragmatic hernia; *ARSA*: aberrant right subclavian artery; *TGA*: complete transposition of great arteries; *RAOA*: right aortic arch; *SUA*: single umbilical artery; *CPC*: choroid plexus cyst; *RPD*: right pulmonary agenesis or dysplasia; *HN*: hydronephrosis; *ANB*: absent nasal bone; *FE*: fetal edema; *AHP*: abnormal hand posture; *TRAPs*: twin reversed arterial perfusion sequence; *R-MCDK*: right polycystic dysplasia kidney; *FGR*: fetal growth restriction

**Table 4:** Frequency of UPSVS coexistent defects/ anomalies

Coexistent defects/ anomalies	Type I n (%)	Type II n (%)	Type IIIA n (%)	Type IIIB n (%)
Central nervous	-	-	-	2 (22.2)
Facial	-	1 (10.0)	-	1 (11.1)
Thorax	1 (25.0)	-	1 (33.3)	-
Cardiovascular	1 (25.0)	3 (30.0)	1 (33.3)	1 (11.1)
Cardiac insufficiency	2 (50.0)	1 (10.0)	-	-
Urinary	-	-	2 (66.7)	-
Limbs	-	1 (10.0)	-	1 (11.1)
Umbilical	1 (25.0)	-	1 (33.3)	1 (11.1)
Edema	-	-	-	1 (11.1)
Growth restriction	-	1 (10.0)	-	1 (11.1)
Chromosome abnormalities	-	4 (40.0)	-	5 (55.6)

“-”: without the defects/ anomalies, without chromosome abnormalities (including the cases for which karyotyping and CNV-Seq were not performed)

## Discussion

### Incidence rate of UPSVS

UPSVS is a low-incidence congenital vascular malformation. The incidence rate in neonatal period is reported to be 1/30,000–1/2,5000 [1, 5]. The present studies on UPSVS are mostly case reports or retrospective studies based on small sample size, and most of the study patients are children or adults [4, 5, 7–9, 15–19]. The incidence rate of UPSVS in fetal period has not been clearly reported. The study by Yagel, et al. that consisted of high- and low-risk groups did not draw conclusion about the prevalence of UPSVS [13]. Studies by Achiron, et al. believed that the incidence rate of fetal UPSVS was unknown [1, 3]. In our study, 24 of the 26 cases of UPSVS were found in 14,261 pregnant women who were referred to our center (Yunnan Provincial Prenatal Diagnosis Center) for ultrasonography due to high-risk in prenatal screening or other prenatal abnormal findings in primary hospitals. Only two cases were found in 18,713 pregnant women who lives in the city of Kunming and accepted routine prenatal care in our hospital. Based on the data, it is estimated that the incidence rate of fetal UPSVS in ordinary pregnancies in the city of Kunming is about 1/9357, which is significantly higher than that reported in prior studies. Some studies suggested that the reason why UPSVS had been less reported prenatally might be the low awareness of this malformation among medical staff [1, 19]. Therefore, raising awareness to UPSVS in prenatal screening is one of the aims of our study.

### Ultrasonographic characteristics and prognosis of fetal UPSVS

This study followed the clinical-anatomical classification of UPSVS put forward by Achiron, et al., which is more in line with the anatomical and hemodynamic changes of UPSVS, and is more conducive to guiding prenatal consultation and postnatal intervention [1]. Despite the low incidence of UPSVS, we have collected considerable cases of fetal UPSVS that covered all types proposed in Achiron’s classification because of the large number of high-risk cases referred from primary hospitals to our center. The results of our study have similar views as Achiron, et al. but also new findings.

Achiron, et al. found that the USS group (type I) had poorest prognosis, lowest live birth rate, and earliest gestational age at diagnosis [1]. The four cases of USS in our study showed that fetal umbilical vein ran on the liver surface and directly connected to the right atrium through a large vein. The left portal vein-ductus complex was not detected in the liver, and the intrahepatic intrinsic portal vein was unclear on ultrasound. All four cases manifested heart enlargement and heart insufficiency at 22–24 gestational weeks. In fetus with USS, the blood flow of umbilical vein does not enter liver but directly enter right atrium, resulting in increased right atrial volume load in earlier stage of gestation, and consequently the fetuses manifested as heart enlargement, heart failure, fetal hydrops, and even fetal death [5].

In Achiron’ study, DVSS group (type II) was the only group in which chromosomal aneuploidy was found [1]. In our study, four of the ten fetuses with DVSS were trisomy 21. One case was a naturally conceived monochorionic diamniotic twin, in which fetus A was type II UPSVS and fetus B was acardia malformation. One case had complicated malformations of fetal edema, NT thickening and cervical cystic hygroma. These two cases declined invasive prenatal diagnosis and chose to terminate pregnancies. The other four cases were isolated type II and had normal karyotype and CNV-Seq results. These four babies were normal in growth and development after birth. About 21% (4/19) of DVSS were trisomy 21 in Achiron’s study, and 44% (4/9) of DVSS were trisomy 21 in our study. In DVSS fetuses with normal karyotype and CNV-Seq results, if the ductus venous connects to subphrenic vestibule other than connecting to right atrium via abnormal veins, leading to increased right heart volume load and consequent fetal edema, the closure of ductus venous to form ligamentum venosa after birth will result in a good postnatal prognosis. If signs of heart insufficiency appear, close ultrasound monitoring is required to assess fetal heart function and to terminate pregnancy in a timely manner. The finding of our study is in accordance with Achiron, et al. that type II had the highest incidence of chromosomal aneuploidy [1].

Achiron’s study suggested that IHPSS group (type IIIA) was characterized by the highest incidence of FGR [1]. In our study, FGR was not found in all three cases of IHPSS. One case was terminated at 25 gestational weeks due to multiple malformations. One case was terminated at 26 gestational weeks due to the compound malformation of right polycystic dysplastic kidney. One case was terminated at 27 gestational weeks due to the appearance of fetal heart failure. The gestational weeks at termination for all three cases were less than 28 weeks. Thereby, whether type IIIA mostly manifest as late-onset FGR needs further studies.

In our study, two cases of trisomy 18 were found in type IIIB. Song, et al. also reported a case of type IIIB diagnosed as trisomy 18 [20]. These findings are inconsistent with Achiron’s finding that type II (DVSS) was the only type that had chromosomal aneuploidy. In our study, seven of nine EHPSS cases terminated pregnancies due to chromosomal abnormalities or (and) complicated structural malformations, and two cases of isolated EHPSS continued pregnancy to term delivery, and were normal in growth and development after birth. The prognosis of fetal EHPSS depended on the development of intrahepatic portal vein and its branches. Cases with complete absence of intrahepatic portal vein (type I EHPSS) had poor prognosis, and liver transplantation could be the only treatment option after birth [6]. For type II EHPSS, portosystemic shunt closure or ligation and hepatic lobectomy can be applied after birth according to the diameter of shunt and the clinical manifestations. In most cases, symptoms will be improved significantly after surgery, but a small number of cases still had pulmonary hypertension, hepatic encephalopathy and intestinal congestion [7].

EHPSS, also known as Abernethy malformation, was first described by surgeon Abernethy in 1793 [18]. Morgan, et al. classified EHPSS into two types according to clinical manifestations and portal vein perfusion: absence of intrahepatic portal vein perfusion (Abernethy type I) and presence of intrahepatic portal vein perfusion (Abernethy type II) [17]. The classification is based on EHPSS after birth. Fetal liver has triple blood supply from umbilical vein, portal vein and hepatic artery other than dual blood supply from portal vein and hepatic artery after birth [11]. Due to the existence of umbilical vein blood supply in fetal period and the absence of umbilical vein blood supply after birth, at present, it is still unknown whether Abernethy type II diagnosed in fetal period transform into Abernethy type I after birth. In prenatal ultrasonography, it is difficult to observe hypoplastic and hypoperfused portal vein and its branches by both 2D ultrasonography and color Doppler [1, 10]. Therefore, fetal EHPSS was not further classified in our study. It is necessary to clearly diagnose the types of Abernethy malformation after birth by angiography which is the gold standard for the diagnosis of Abernethy malformation [9].

### Prenatal ultrasonographic techniques of assessing UPSVS

Prenatal diagnosis of UPSVS is depending on the sonographers’ experiences. When absence or dysplasia of portal vein branches, broadened ductus venosus and/ or hepatic vein are observed, UPSVS should be suspected, and umbilical vein, ductus venosus, and intrahepatic and extrahepatic portal veins need to be examined in details.

Prenatal diagnosis of Type I is not difficult. It is easy to identify widened umbilical vein that confluences into right atrium or inferior vena cava. In type II, when ductus venosus is found not entering subphrenic vestibule, we will look for the route and inlet of ductus venosus and if it flows into hepatic segment of inferior vena cava or hepatic vein, thereby, diagnosis of Type II can be made. In type IIIA, sometimes there are widened communicating vessels, and even hemangiomas, between intrahepatic portal vein and hepatic vein. In these case, diagnosis is easily made. However, in some cases, the communicating vessels are too small to be identified, so that the diagnosis become difficult. In type IIIB, no portal vein trunk confluences into portal sinus at the first porta hepatis. Due to the inadequacy of blood flow, dysplastic intrahepatic portal vein, especially the right portal vein and its branches are often observed by experienced sonographers. However, superior mesenteric vein and splenic vein are small in fetal stage, merely 1 ~ 2 mm in diameter. It is difficult to further classify type IIIB. Application of such techniques as 3D HD-live flow, 4D, STIC, Slow flow can be helpful.

We summarized the procedures for diagnosis of UPSVS as “five-plane method”: (1) scan the junction of umbilical vein and portal vein at the abdominal circumference transverse section, (2) scan main portal vein and its branches at abdominal transverse section, (3) scan the confluence of superior mesenteric vein and splenic vein where main portal vein forms, (4) scan the confluence of left, middle and right hepatic veins into inferior vena cava at abdominal circumference transverse section, (5) scan the reflux routes of umbilical vein, ductus venosus and inferior vena cava at parasagittal section of upper abdomen.

### Limitations of this study

This study had limitations. Firstly, since this was a retrospective study, we had neither confirmed prenatal ultrasonographic findings at the time after abortion nor collected fetal specimen for later fetopsy. However, we have started this work recently, and our future studies on fetal structural anomalies will be improved. Secondly, as we are a provincial prenatal diagnosis center, most of the cases enrolled in this study were transferred from local hospitals, and they also delivered in local hospitals later. Confirmation of prenatal findings after birth, and physical examination, genetic evaluation and postnatal imaging for the children by our center would be time-consuming and labor-intensive. Therefore, we merely learned about the postnatal situation of these live-birth children through telephone follow-up. Fortunately, all of the 6 children had postnatal reexamination and regular pediatric health check in local hospitals, which preliminarily confirmed that they were normal. Finally, ultrasound diagnosis of UPSVS is depending on the sonographers’ experiences. Prenatal ultrasonography in local hospitals might miss some cases of UPSVS, which could lead to an underestimation of incidence rate of UPSVS in our study.

To sum up, fetal UPSVS can be clearly diagnosed by prenatal ultrasonography, and be typed according to Achiron’s classification method that reflects the anatomical and hemodynamic changes of the malformation, which is more conducive to guiding prenatal consultation and postnatal intervention. The incidence of chromosomal abnormalities in fetuses with UPSVS is related to the types of UPSVS. When fetal UPSVS complicates chromosomal abnormalities or other structural malformations, the fetal prognosis is poor. Due to the absence of portal vein which leads to hepatic ischemia and secondary hepatic encephalopathy, some neonates with Abernethy type I may die before a clear diagnosis [10]. Therefore, prenatal diagnosis and classification of UPSVS, and invasive prenatal diagnosis to exclude chromosomal abnormalities, are of great clinical significance for the assessment of fetal prognosis and early intervention for affected neonates. The number of cases in our study is still inadequate, and the follow-up time after birth is insufficient. It is necessary to collect more cases and follow up for longer time in the future studies to provide more valuable information for prenatal consultation and postnatal management.

## Data Availability

The datasets used and/or analyzed during the current study available from the corresponding author on reasonable request.

## References

[1] Achiron R, Kivilevitch Z (2016). Fetal umbilical-portal-systemic venous shunt: in-utero classification and clinical significance. Ultrasound Obstet Gynecol.

[2] Engelbrechtsen L, Brøndum-Nielsen K, Ekelund C, Tabor A, Skibsted L (2013). Detection of triploidy at 11–14 weeks’ gestation: a cohort study of 198 000 pregnant women. Ultrasound Obstet Gynecol.

[3] Achiron R, Gindes L, Gilboa Y, Weissmann-Brenner A, Berkenstadt M (2010). Umbilical vein anomaly in fetuses with Down syndrome. Ultrasound Obstet Gynecol.

[4] Wang Y, Wei J, Liu G, Pei Q (2018). Prenatal diagnosis and counselling for congenital portosystemic venous shunts. Chin J Perinat Med.

[5] Dauvillée J, Ingargiola I, Jouret M, Biard JM, Steenhaut P, Bernard P (2020). Fetal umbilical-systemic shunt with a positive issue. J Gynecol Obstet Hum Reprod.

[6] Zhao L, Wu L (2020). Diagnosis and management of congenital portosystemic shunts in children. Chin J Pediatr Surg.

[7] Xiao Y, Zeng Y, Xiao Z, Li W (2019). Clinical analysis of pulmonary arterial hypertension associated with congenital portosystemic shunt in children. J Clin Pediatr.

[8] Xie Y, Zhao B, Liu Y, Wang C. Congenital portosystemic shunts in infant with different clinical symptoms: Two cases report. *Chinese J Medical Imaging Technol* 2019, 12:1927.

[9] Shen G-y, Jiang X-j (2018). A case report of congenital extrahepatic portosystemic venous shunt—abernethy malformation and literatures review. Chin J Liver Dis.

[10] Shen O, Valsky DV, Messing B, Cohen SM, Lipschuetz M, Yagel S (2011). Shunt diameter in agenesis of the ductus venosus with extrahepatic portosystemic shunt impacts on prognosis. Ultrasound Obstet Gynecol.

[11] Zeng H, Tang S, Qin S, Wang X (2020). Progress in the clinical diagnosis and treatment of hepatic vascular diseases. Chin J Hepatol.

[12] Ultrasound Branch of Chinese Doctors Association: Chinese Guidelines for prenatal ultrasound. (2012). *Chinese Journal of Medical Ultrasound* 2012, 7:4–10.

[13] Yagel S, Cohen SM, Valsky DV, Shen O, Lipschuetz M, Messing B (2015). Systematic examination of the fetal abdominal precordial veins: a cohort study. Ultrasound Obstet Gynecol.

[14] Li Y, Fang J, Zhou K, Wang C, Hua Y, Shi X, Mu D (2015). Prediction of fetal outcome without intrauterine intervention using a cardiovascular profile score: a systematic review and meta-analysis. J Matern Fetal Neonatal Med.

[15] Ji M, Wu X, Gong Y, Yang B, Qiao Z (2017). Imaging features of 8 cases of congenital portosystemic shunt with gastrointestinal hemorrhage: case series report. Chin J Evidence-Based Pediatr.

[16] Zhang J, Li L, Li Q, Qiao G, Chen X (2017). Surgical treatment of five cases of Abernethy malformation type II. Chin J Surg.

[17] Morgan G, Superina R (1994). Congenital absence of the portal vein: two cases and a proposed classification system for portasystemic vascular anomalies. J Pediatr Surg.

[18] Abernethy J. Account of Two Instances of Uncommon Formation in the Viscera of the Human Body: From the Philosophical Transactions of the Royal Society of London. Med Facts Obs. 1797;7:100–108. PMC5111139 29106224

[19] Han BH, Park SB, Song MJ, Lee KS, Lee YH, Ko SY, Lee YK (2013). Congenital portosystemic shunts: prenatal manifestations with postnatal confirmation and follow-up. J Ultrasound Med.

[20] Song Y, Song Z, Sun L, Chen T (2018). Detection of trisomy 18 with Abernethy malformation by prenatal ultrasound: a case report. Chin J Perinat Med.

